# Video-Game Play Induces Plasticity in the Visual System of Adults with Amblyopia

**DOI:** 10.1371/journal.pbio.1001135

**Published:** 2011-08-30

**Authors:** Roger W. Li, Charlie Ngo, Jennie Nguyen, Dennis M. Levi

**Affiliations:** 1 School of Optometry, University of California, Berkeley, California, United States of America; 2 Helen Wills Neuroscience Institute, University of California, Berkeley, California, United States of America; Bremen University, Germany

## Abstract

Abnormal visual experience during a sensitive period of development disrupts neuronal circuitry in the visual cortex and results in abnormal spatial vision or amblyopia. Here we examined whether playing video games can induce plasticity in the visual system of adults with amblyopia. Specifically 20 adults with amblyopia (age 15–61 y; visual acuity: 20/25–20/480, with no manifest ocular disease or nystagmus) were recruited and allocated into three intervention groups: action videogame group (*n* = 10), non-action videogame group (*n* = 3), and crossover control group (*n* = 7). Our experiments show that playing video games (both action and non-action games) for a short period of time (40–80 h, 2 h/d) using the amblyopic eye results in a substantial improvement in a wide range of fundamental visual functions, from low-level to high-level, including visual acuity (33%), positional acuity (16%), spatial attention (37%), and stereopsis (54%). Using a cross-over experimental design (first 20 h: occlusion therapy, and the next 40 h: videogame therapy), we can conclude that the improvement cannot be explained simply by eye patching alone. We quantified the limits and the time course of visual plasticity induced by video-game experience. The recovery in visual acuity that we observed is at least 5-fold faster than would be expected from occlusion therapy in childhood amblyopia. We used positional noise and modelling to reveal the neural mechanisms underlying the visual improvements in terms of decreased spatial distortion (7%) and increased processing efficiency (33%). Our study had several limitations: small sample size, lack of randomization, and differences in numbers between groups. A large-scale randomized clinical study is needed to confirm the therapeutic value of video-game treatment in clinical situations. Nonetheless, taken as a pilot study, this work suggests that video-game play may provide important principles for treating amblyopia, and perhaps other cortical dysfunctions.

**Trial Registration:**

ClinicalTrials.gov NCT01223716

## Abstract 2

A pilot study suggests that playing video games may enhance a range of spatial vision functions in adults with amblyopia.

## Author Summary

Early abnormal visual experience disrupts neuronal circuitry in the brain and results in reduced vision, known as amblyopia or “lazy eye,” the most frequent cause of permanent visual loss in childhood. It is generally believed that adult amblyopia is irreversible beyond the sensitive period of brain development during childhood. In this study, we examine whether playing video games, both action and non-action, has an effect on the vision of adults with amblyopia. We assessed visual acuity (visual resolution), positional acuity (the ability to localize object's relative position), spatial attention (the ability to direct visual attention to various locations in the visual field), and stereoacuity (stereo-vision / 3-D depth perception) in a small group of teenagers and adults. We found that they tended to recover vision much faster than we would have expected from the results of conventional occlusion therapy in childhood amblyopia. Additional experiments and modelling suggest that the improvements are a result of decreasing spatial distortion and increasing information processing efficiency in the amblyopic brain. Thus, video games may include essential elements for active vision training to boost visual performance. Most importantly, our findings suggest that video-game play may provide important principles for treating amblyopia, a suggestion that we are pursuing with larger scale clinical trials.

## Introduction

The most frequent cause of permanent visual loss in childhood is amblyopia (“lazy eye”) [1],[2], a developmental disorder associated with early abnormal visual experience that disrupts neuronal circuitry in the visual cortex and results in abnormal spatial vision. It is generally believed that adult amblyopia is irreversible beyond the sensitive period of brain development. However, new studies, both in humans [3]–[12] and in rodents [13]–[15], suggest that the mature amblyopic brain retains a substantial degree of plasticity. In particular, human adults with long-standing amblyopia show substantial improvements in performing a visual task, following perceptual learning (extended practice) of the task.

Playing video games results in enhancement of a broad range of visual tasks in adults with normal vision, including light sensitivity [16], contrast sensitivity [17], visual crowding [18], and visual attention [19]. However, while it is now clear that video-game play can strengthen some aspects of normal vision, it is not clear whether video-game play can induce functional plasticity in the mature visual system following a prolonged period of abnormal development. Moreover, while video-game play improves the spatial resolution of attention in normal participants, it does not improve visual acuity (with isolated targets). Since reduced visual acuity is the sine qua non of amblyopia, it is crucial that video-game play can improve visual acuity if it is to be a useful tool for visual rehabilitation in patients with reduced spatial vision.

In the present study, we aimed to assess with a small pilot group whether playing video games with an amblyopic eye can induce cortical plasticity and improve spatial vision in adults with amblyopia, well beyond the “sensitive period” of brain development. We hypothesized that the intense sensory-motor interactions while immersed in video-game play might push brain functions to the limit, enabling the amblyopic visual system to learn, on the fly, to recalibrate and adjust, providing the basis for functional plasticity. Moreover, game playing requires the allocation of spatial attention, detection, and localization of low contrast, fast moving targets, and aiming (in first-person shooter games). Thus, we speculated that video games may include several essential elements for active vision training to boost visual performance, and thus could potentially be useful in improving amblyopic vision.

**Figure 1:**
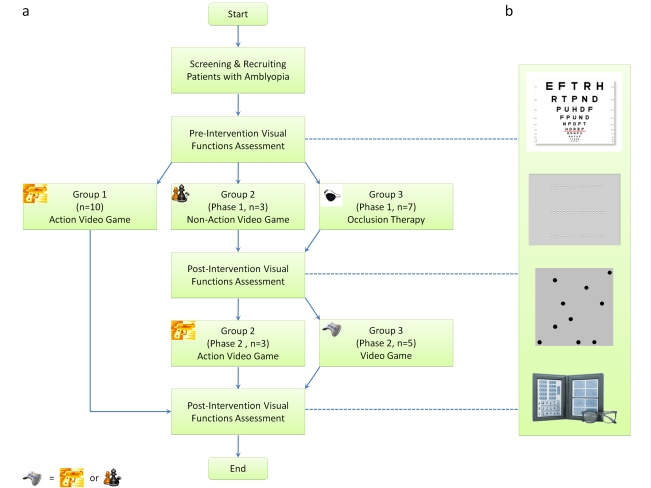
Consort flow diagrams. This research project was commenced in late 2004 and completed in early 2009. The first author (RWL) was responsible for conducting clinical procedures in screening patients and assigning participants to interventions. Participants were pseudo-randomly allocated into three intervention groups. The first 10 enrolled patients participated in the action videogame group (MOH), the subsequently enrolled three patients participated in the non-action videogame group (SIM), and then another seven patients were recruited in the crossover intervention group (phase 1: occlusion therapy; phase 2: video game therapy, “joypad” symbol  =  MOH or SIM). Note that the subject allocation was not based on the clinical characteristics of participants.

We tested a range of visual functions to examine the neural alternations, if any, following video-game play in a small group of adults (Figure 1). These visual functions, ranging from low-level to high-level vision, included visual acuity (letter acuity), positional acuity (Vernier acuity), visual counting (spatial attention), and stereoacuity (3-D binocular vision). In order to understand the neural mechanisms that underlie the video-game experience induced visual plasticity, we measured and modeled a positional acuity task in noise.

While action video games are reported to be useful in enhancing visual function in normal humans, non-action video games are not effective [19]. Playing action video games may not be ideal for patients with amblyopia, particularly children. Therefore, in another set of pilot experiments, we also examined whether non-action video games may be effective for recovering amblyopic visual functions.

Our participants played video games for 40 h with their fellow eye patched. One might argue that the visual improvements, if any, might have resulted from the eye patching alone. To address this point, we used a cross-over treatment design in which a group of amblyopes first underwent occlusion therapy, i.e. patching the fellow sound eye, for a period of time before the video-game phase. With this experimental design, we can compare the efficacy of the two treatment approaches (passive patching and video-game playing).

Our study had several limitations: small sample size, lack of randomization, and differences in numbers between groups. A large-scale randomized clinical study is needed to confirm the therapeutic value of video-game treatment in clinical situations. Nonetheless, taken as a pilot study, this work suggests that video-game play may help guide future treatment of amblyopia.

## Results

To evaluate how video-game play alters amblyopic vision, we monitored the changes, if any, in visual acuity in 10 adults with amblyopia while they played a first-person shooter game—Medal of Honor: Pacific Assault (MOH)—using their amblyopic eye, with the fellow sound eye patched with a black eye patch. Visual acuity (VA) is a standard clinical procedure to quantify spatial vision by determining the smallest letter on a chart that can be identified at a given viewing distance. In amblyopia, vision is often substantially poorer when the target letter is presented with surrounding letters than when it is presented alone, a phenomenon known as crowding [20]. Therefore we measured both crowded line-letter acuity and uncrowded single-letter acuity so as to provide a comprehensive evaluation of visual acuity.

**Figure 2:**
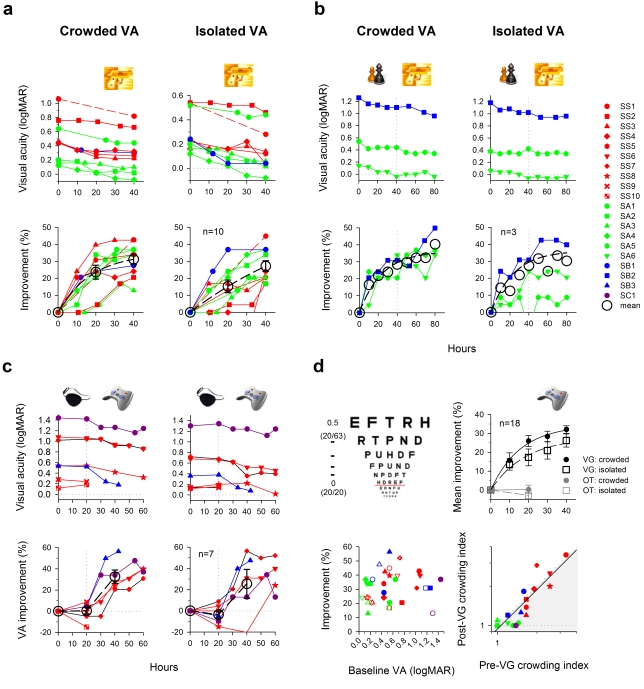
Improved visual acuity (VA) with video-game experience. (A) Action video game. Color coding is used throughout the figures to represent the type of amblyopia. Red, strabismic; green, anisometropic; Blue, mixed (strabismic & anisometropic); dark purple, mixed (strabismic & deprivation). Error bars: one s.e.m. (here and in all subsequent figures). (B) Non-action video game. In this experiment, participants were required to play a non-action video game (“chess” symbol: SIM) in the first 40 h and an action video game (“gun” symbol: MOH) in the second 40 h. Note that given the small sample size, the fitting curve is provided here for reference. (C) Control experiment. Another group of participants was required to first undertake occlusion therapy (OT, “patch” symbol) for 20 h, and then continue to the video-game phase (“joypad” symbol: MOH or SIM). Note that SB3 was not available to finish the complete course of video-game training. (D) Summary of acuity data. (Top left) A schematic logMAR letter chart. Each 0.1 logMAR represents 1 letter-line. Parentheses: Snellen acuity. (Top right) The visual acuity data from panels a–c are pooled together to calculate the mean data. (Bottom left) Percent improvement is replotted as a function of baseline visual acuity. Solid symbols: crowded acuity. Open symbols: uncrowded acuity. (Bottom right) Effect of video-game experience on visual crowding. Shaded area: decreased visual crowding.

Surprisingly, playing video games rapidly reversed their amblyopia. After 40 h of video-game play (2 h/d), acuity improved, on average, by 1.6 and 1.4 lines on a LogMAR letter chart for crowded letters and single letters, respectively (Figure 2a, top panels), representing 31.2%±3.1% (crowded: *t* = 10.154, *p*<0.0001) and 27.2%±3.2% (uncrowded: *t* = 8.598, *p*<0.0001) improvements in minimum angle of resolution (MAR—bottom panels). Two mild amblyopes (SA2 & SA4) completely “normalized” according to a criterion of 20/20 (LogMAR = 0, dotted line). It might be argued that the improvements could be due to learning the letter charts. Therefore, instead of taking measurements every 10 h, we tested observer SS1's acuity only before and after the video-game intervention, and similar to what we observed in other observers, his acuity improved substantially (≈2.5 letter-lines or ≈44% for both measurements).

While it has been clearly demonstrated that playing action video games improves a broad array of visual functions in adults with normal vision, non-action games are not effective [17],[18]. For example, playing action video games resulted in enhanced crowded resolution acuity in normal vision, while playing a non-action video game did not. However, action games may not be ideal for patients with amblyopia, particularly younger patients. Therefore, in the next experiment, we asked another three amblyopic patients to play a non-action video game—SimCity Societies (SIM).

Interestingly, we found that similar to the action game group, all three non-action game players showed enhanced vision (Figure 2b, phase 1: 0 to 40^th^ h), and one, a mild amblyope (SA6) normalized to ≈20/16. On average, this group was able to read 1.5 more letter-lines (28.4% improvement) for crowded-letter acuity and 0.8 more lines (15.1% improvement) for single-letter acuity. These findings suggest that non-action games share useful properties for enhancing amblyopic vision. To determine the limits of plasticity, the three players who participated in the SimCity experiments were then asked to play MOH for another 40 h (phase 2: 40^th^ to 80^th^ h). Additional improvements of about one letter-line (SB2 & SA5, crowded: 18%) were observed. Note that SA6's amblyopia was completely normalized at the end of phase I and no further significant improvement was observed.

Since our participants played video games with the fellow eye patched, the vision enhancement we observed could have been the result of wearing an eye patch alone. Thus, in a control experiment, another group (OT) of seven amblyopic adults wore a patch, but instead of playing video games they were required to engage in other visually demanding activities, such as watching television, reading books, knitting, and surfing the Internet, using the amblyopic eye. After 20 h, however, no significant change in acuity was observed (Figure 2c, phase 1: 0–20^th^ h); the dashed line in the bottom panels shows the mean data (OT20: crowded: mean improvement = 0.4%±3.0%, *t* = 0.1317, *p* = 0.8995; uncrowded: mean improvement = −3.7±3.2%, *t* = 1.136, *p* = 0.2991). In contrast, for the same amount of time, the video-game group (*n* = 9) showed a marked improvement in acuity of ≈20% (Figure 2a, MOH20>OT20: crowded: *t* = 4.337, *p* = 0.0007; uncrowded: *t* = 3.74, *p* = 0.0022). Five of the seven participants who completed the patching experiment continued to the video-game phase for another 40 h. In this phase of the experiment, we used both action (all except SC1) and non-action (SC1) games. Although none of the five showed any significant change in acuity in the patching phase, all improved substantially in the video-game phase (OT-VG20: ≈1.7 letter-lines, ≈29% improvement in both measurements; OT20<OT-VG20: crowded: paired *t* = 5.712, *p* = 0.0046; uncrowded: paired *t* = 2.785, *p* = 0.0495). From this small-scale “cross-over” experimental design, we can conclude that it is the video-game experience, and not simply the patching, that enhances amblyopic vision.

Figure 2d summarizes all the acuity data from the above experiments. The mean improvement in visual resolution across all 18 participants who completed the video-game training from the three experiments was ≈30% (crowded acuity: 1.8 letter-lines, 33.4%±2.4% and isolated acuity: 1.5 letter-lines 27.4%±3.5%). The effect sizes (Cohen's *d* value) at the 20^th^ h were 3.03 and 1.33 for crowded acuity and isolated acuity, respectively. The recovery of crowded acuity was slightly faster than uncrowded acuity. An exponential fit *y* = *y_o_*+*a*(1−*e* ^−*bx*^) to the data revealed time constants (*b*) of 0.064 and 0.054 h^−1^ for crowded acuity and uncrowded acuity, respectively. It is worthwhile noting that the recovery rate we observed here in adults is ≈5-fold faster when compared with the conventional occlusion therapy in children. It would take >200 h to obtain comparable treatment effects in children (≈0.1 logMAR unit/120 h) [21], and it would be reasonable to expect a much longer treatment course for adults [22].

There was no significant correlation between the amount of acuity improvement and the baseline acuity (Figure 2d bottom left). The mean crowding index, crowded acuity (MAR) / uncrowded acuity (MAR), was slightly, but not significantly, reduced (by 5.9%±4.3%), indicating that video-game play improved crowded acuity slightly more than uncrowded acuity (Figure 2d bottom right).

**Figure 3:**
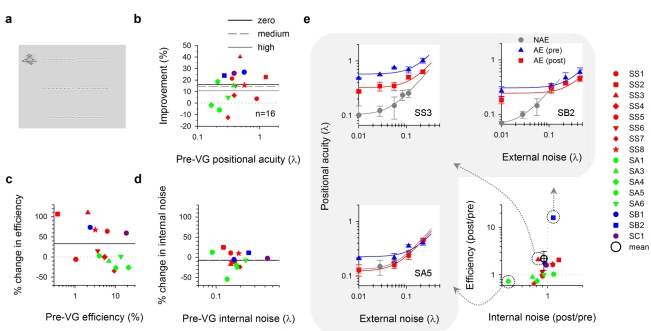
Improved positional acuity with video-game experience. (a) Position discrimination. The visual task was to pick the misaligned pair of Gabor patch groupings out of three choices (top, middle, or bottom) [23]. Each grouping consisted of 8 Gabor patches. Positional noise to the Gabor patches was introduced by varying their vertical positions according to a Gaussian distribution function. (b) Percent improvement in positional acuity as a function of baseline positional acuity (zero noise). Each data point represents the mean improvement across different noise levels. (c) Effect of video-game experience on sampling efficiency. (d) Effect of video-game experience on internal noise. (e) Threshold versus noise (TvN) function. Three different neural mechanism signature profiles are illustrated. SB2: TvN function shifts downward (increase in efficiency). SA5: The knee point of TvN function shifts downward and to the left (decrease in internal noise). SS3: combination of both.

While visual acuity represents one important limit to spatial vision, positional acuity, which represents the ability to localize visual objects, is another important aspect of spatial vision. While positional acuity is remarkably acute in normal vision (often referred to as hyperacuity), it is often severely impaired in amblyopia. We found that positional acuity (the ability to detect a misalignment between the two line segments—Figure 3a) improved significantly following video-game play (on average 16.0%±4.0%; *n* = 16 [MOH40: 12+SIM40: 4]—Figure 3b, black solid line, zero external noise: *t* = 3.963, *p* = 0.0012; non-amblyopic eye: 1.0%±10%, *t* = 0.1057, *p* = 0.9179 [*ns*]).

To understand the neural mechanisms underlying this improvement, we introduced positional noise [23] to mimic the spatial distortions (internal spatial noise) existing in the visual system and applied a positional averaging model to the data (see Materials and Methods). Figure 3c shows that the ability to extract and process information from the visual stimuli for positional averaging was actually boosted by 33.1%, with mean sampling efficiency improved from 6.8% to 9.0%. Some observers also showed reduced spatial distortion (on average internal noise decreased by 7%—Figure 3d, from 0.185 λ to 0.172 λ), indicating that the distorted retinotopic cortical mappings were recalibrated and less distorted. Fig 3e summarizes the different neural mechanisms (SB2: sampling efficiency enhancement; SA5: spatial distortion reduction; SS3: combination of both) that underlie the improvement in positional acuity.

**Figure 4:**
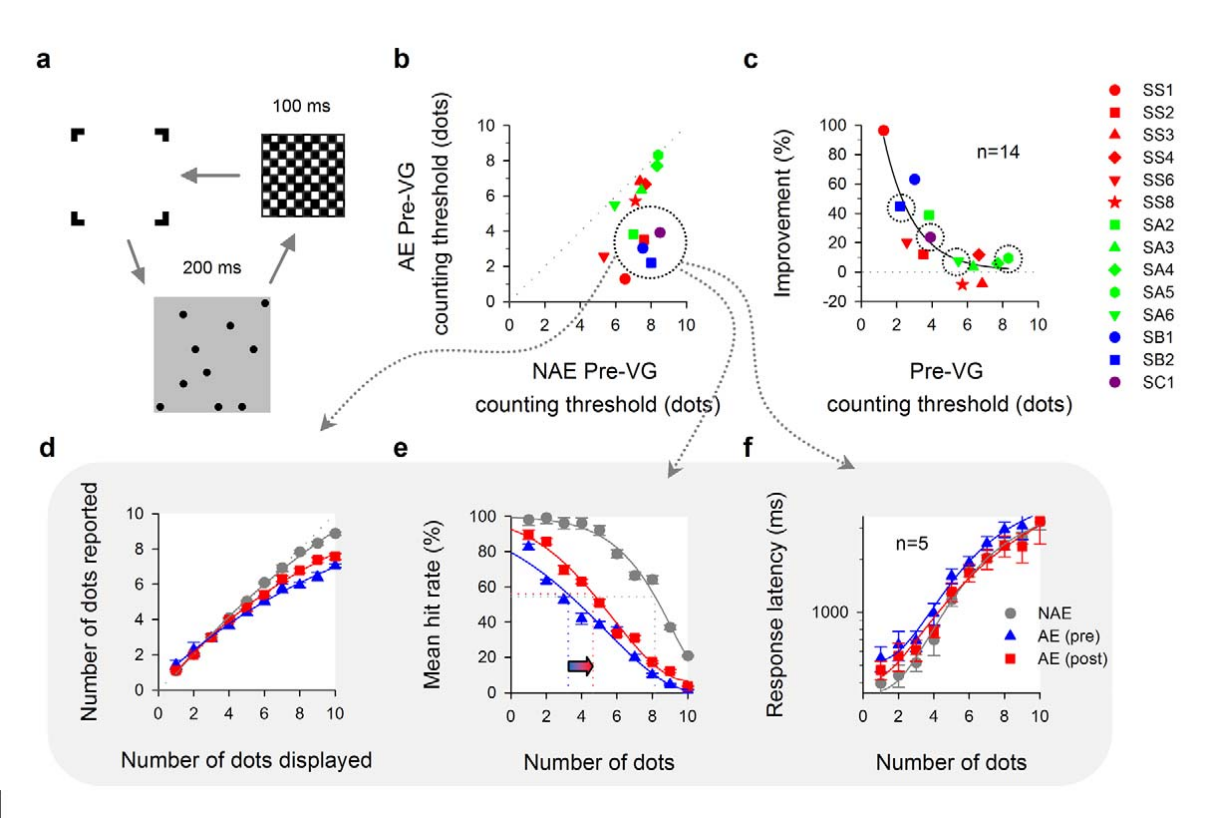
Improved spatial attention with video-game experience. (a) Visual counting. A number (*N* = 1–10 dots) of black circular dots was presented for 200 ms against a gray background. The target stimulus was then followed by a checkerboard pattern for another 100 ms. Observers were asked to enumerate the number of dots as quickly and accurately as they could. No feedback was given. Note that the dot size was scaled with visual acuity, and therefore the dots displayed on the screen were very visible. (b) Counting threshold. Non-amblyopic eye (NAE) versus amblyopic eye (AE). (c) Percent improvement of counting threshold in the amblyopic eye after video-game intervention. SIM: *n* = 4 (dotted circles). MOH: *n* = 10. (d–e) Subgroup analysis—Undercounting. (d) Number of dots reported as a function of number of dots displayed. (e) Counting threshold calculation. An arrow indicates an increase in counting threshold. (f) Response latency as a function of number of dots.

Video-game play also appears to increase visual attention in amblyopia. We used a visual counting task to determine how many visual locations the brain can direct attention to in a very brief time period, 200 ms (Figure 4a). Previous work has shown that some amblyopes show severe deficits in visual counting [24] and that action game play can enhance counting in normal vision [19]. In general, participants who initially showed the largest deficits in counting performance also showed the most improvement (Figure 4c). A subgroup of five participants (symbols surrounded by dotted circles in Figure 4b) showed significant undercounting (Figure 4d, blue line). For example, when 10 dots were displayed, the mean number of dots reported was 7 (undercounted by 3 dots or 30%). Undercounting is thought to reflect high-level neural deficits in amblyopia [24]. Following video-game play, for the range of 7–10 dots, undercounting decreased significantly by 8.4% (from 25.3% to 16.9%—Figure 4d, two-way RM ANOVA: *F* = 33.022, *p* = 0.005; non-amblyopic eye: pre 5.6%→post 5.0%, two-way RM ANOVA: *F* = 0.609, *p* = 0.492 [*ns*]). The mean counting threshold (the number of dots that can be reliably counted) increased significantly by 37%, from 3.3±0.3 to 4.4±0.4 dots (Figure 4e, paired *t* = 4.508, *p* = 0.0108; non-amblyopic eye: pre 7.7±0.3 dots→post 8.0±0.3 dots, paired *t* = 1.161, *p* = 0.3102 [*ns*]) and the mean response latency decreased by 16.5% (Figure 4f, *N* = 1–10), though not significantly (two-way RM ANOVA: *F* = 0.839, *p* = 0.424). In short, video-game play increases the number of items the amblyopic brain can direct attention to simultaneously, reduces undercounting deficits, and increases the processing speed of visual counting.

**Figure 5:**
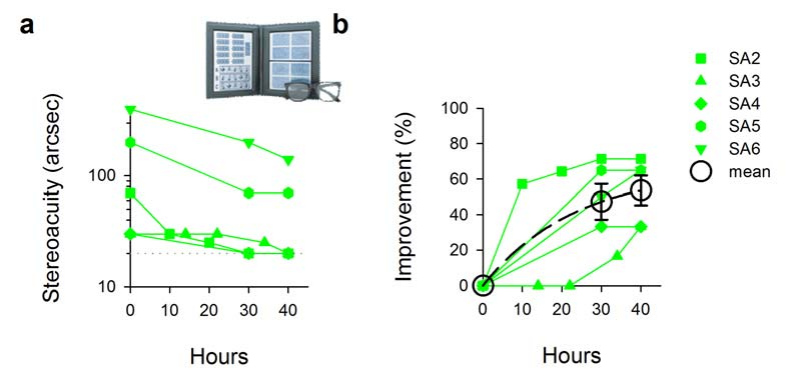
Improved stereoacuity in anisometropic amblyopia with video-game experience. (a) Stereoacuity as a function of video-game hours. The normal stereoacuity range is 20–40 arcsec. Dotted line: the lower measuring limit of the stereo test plates. Note: JS failed the test in the baseline session; her initial data point is thus arbitrarily set to 400 arcsec (the upper measuring limit of the test plates). (b) Stereoacuity data were replotted in terms of percent improvement.

Amblyopia is associated with abnormal binocular vision and reduced or absent stereopsis (binocular depth perception or 3-D). With improved monocular vision following video-game play, for some amblyopes binocular vision also recovered to a substantial extent. Five of the six anisometropic amblyopes (with straight eyes) were tested for stereopsis following the training. All five showed improved stereopsis (Figure 5a, *n* = 5 [MOH40: 3+SIM40: 2]). Mean improvement in stereoacuity was 53.6%±8.4% (Figure 5b, *t* = 6.410, *p* = 0.003), noting that SA6 failed the stereo test and had no recordable stereopsis in the baseline session. Three participants (SA2, SA3, and SA4) fully regained normal stereoacuity (20 arc sec) as measured by this test, and were basically “cured” in this aspect of vision.

## Discussion

Here we provide evidence from a pilot study of a small number of people that video-game play can induce a substantial degree of visual plasticity in adults with amblyopia. After a brief period of video-game play, a wide range of spatial vision functions improve very rapidly and substantially, reflecting normalization of both low-level (visual acuity, positional acuity) and high-level (spatial attention, stereoacuity) visual processing. Importantly, we provide preliminary characterization of the time course, limits, and underlying mechanisms of video-game experience-dependent cortical plasticity. The findings of our “cross-over control” experiment show that the treatment effects cannot be simply explained by eye patching, suggesting that it is indeed the video-game experience which improves amblyopic vision.

The visual plasticity stimulated through video-game training has been well documented in the “normal” visual system, however the neural mechanisms are not yet clear. Using positional noise, we are able to reveal the underlying mechanisms. As we previously reported, repeatedly practicing a Vernier task in positional noise, with response feedback, can improve sampling efficiency and re-calibrate the distorted retinal topographical mappings of the amblyopic visual field [11],[17]. Here we show that video-game play also results in a substantial increase in the ability to extract visual information (increased sampling efficiency), without specific direct training, and we found that spatial distortion (or internal positional noise) can also be reduced to a certain extent through video-game play. Our findings also provide insights into which levels of processing in visual cortex can be modified. Counting deficits in amblyopes are thought to reflect a higher level limitation in the number of features (and missing features) the amblyopic visual system can individuate [24]. We speculate that the reduction in undercounting deficits in our amblyopic participants may represent the normalization of these higher level cortical areas. Recent work suggests that the ability to apprehend numbers may reflect a primary sensory attribute [25], possibly reflecting the responses of neurons in parietal cortex that are tuned to number. From this perspective, the response characteristics of these affected numerosity processing neurons might be modifiable with video-games experience. While it is possible that low-level factors such as crowding [26] may result in improved counting in amblyopes, we can safely exclude this cause since our observers did not show any significant recovery in crowding. Our regression analysis suggests that changes in crowded acuity account for 3% of the variance in counting threshold and changes in isolated acuity account for 77%.

Perhaps most importantly, we show that playing video games can indeed improve visual acuity and sharpen amblyopic vision. Note that visual acuity is the gold standard for examining spatial vision in clinical situations. To our knowledge, our work is the first to report that uncrowded visual acuity can be improved through video-game training. Green and Bavelier [18] reported that 30 h of video-game play did not result in improved visual acuity in normal adults, perhaps because there is little room for improvement in the normal visual system, or because 30 h is simply not long enough to improve a function as fundamental as normal visual acuity. Here we find that video-game play, both action and non-action, can result in a substantial improvement of amblyopic visual acuity. This is especially important because reduced visual acuity is the sin qua non of amblyopia.

Playing a non-action game for 30 h has been found to be ineffective in enhancing attentional performance in participants with normal vision [19]. However, our results suggest that not only action but also non-action video games might be effective in improving amblyopic spatial vision. Although non-action games do not impose the same intense pressure on the player to respond to sudden pop-up targets from somewhere in the visual field, and to track fast moving objects, they do require the player to pay attention to fine and small spatial details and to different visual features in the visual scene—which may be a very demanding visual task for someone with reduced vision. In fact we noted that during game play, some deep amblyopes initially required more time than normal participants and had to get closer to the screen in order to identify targets or read instructions. In some sense, this is essentially similar to training spatial resolution [27]. A long period of sustained attention in seeing fine visual details might play an important role in triggering neural plasticity. It is worth noting that we had fewer participants, altogether four (three from Group 2 and an extra one from the cross-over group SC1), for the non-action video game. We recognize that the treatment effects could vary from individual to individual. A much larger sample size is necessary for future studies to investigate which type, action or non-action, is more effective in treating amblyopia.

Perceptual learning has shown to be useful in improving amblyopic vision [28]. It is worthwhile noting that the visual recovery, e.g. visual acuity and positional acuity, we observed here with video-game play, although substantial, is somewhat smaller when compared with perceptual learning [4]. However, it is not too surprising that direct training can produce greater improvements, as it usually involves a large number of practice trials (for example, deep amblyopes might need more than 50,000 trials to reach the plateau levels [11]) in which the task difficulty is very challenging, most of the time around the observers' threshold limits. In contrast to perceptual learning, video games provide a visually enriched and stimulating environment, demanding different fundamental visual skills. Animal studies have highlighted the importance of environmental enrichment in promoting cortical plasticity [13],[14]. We postulate that the intense sensory-motor interactions while immersed in video-game play might push brain functions to the limit, enabling the visual system to learn, on the fly, to recalibrate and adjust, providing the basis for functional plasticity.

Treatment of adult amblyopia has recently received considerable attention ever since the introduction of perceptual learning techniques in the past few years [28]–[30]. There have been numerous attempts to find an effective treatment for amblyopia. These attempts include subcutaneous injection of strychnine [31], flashing red and blue lights [32],[33], and rotating gratings [34]. Other more recent studies have attempted to use electric stimulation [35], direct transcranial magnetic stimulation [36], and pharmacological approaches [37] to induce brain plasticity. Some of these techniques seem promising, but the others lack repeatable clinical evidence.

Before a video-game-based approach is used to treat amblyopia clinically, there are still many questions to be addressed (e.g., dose-response, prognosis for different ages of onset, types and depths of amblyopia). The current study serves as a “pilot” trial and, as such, has several design limitations: lack of randomization, small study size, and differences in numbers between arms. The lack of randomization and differences in numbers between arms may have resulted in potentially imbalanced makeup of the study arms on baseline characteristics. For example, the action game group was much more likely to be male and younger than the other groups. In addition, the small number of participants (four) in the non-action game group makes it difficult to draw strong conclusions. A much larger sample size is necessary for future studies to investigate which type, action or non-action, is more effective in treating amblyopia. Specifically, a large-scale randomized double-blind clinical trial (with equal numbers in each group) is needed to eliminate differences between people, placebo effects, and measurement differences. Despite these limitations, the present pilot study provides new insights into how video-game play sharpens visual functions in adult amblyopia and, most importantly, reveals that video-game play may provide important principles for improving treatment in amblyopia, and perhaps other clinical abnormalities.

## Materials and Methods

### Ethics Statement

The experimental procedures were approved by the University Committee for the Protection of Human Subjects, and the research was conducted according to the principles expressed in the Declaration of Helsinki. Informed consent was obtained from each participant. There was no known risk involved in the experimental procedures.

### General Experimental Design

Altogether 20 adults with amblyopia participated in three video-game experiments (age range: 15–61 y, mean age: 31.4±3.5 y). They were recruited through advertisements in newspapers and through the Internet websites. Thorough eye examination was carried out by an experienced optometrist (first author, RWL). Our participant inclusion criteria included: (1) age >15 years; (2) all forms of amblyopia, e.g. strabismic, anisometropic, refractive, deprivative, and meridional amblyopia; and (3) interocular visual acuity difference of at least 0.1 LogMAR. Exclusion criteria included any ocular pathological conditions (e.g., macular abnormalities) and nystagmus. All of our participants had a difference in crowded visual acuity of two lines or more between the two eyes, and had normal vision in the sound eye (∼20/12–20/16). The maculae of all participants were assessed as normal, and they all had clear ocular media (as assessed by direct ophthalmoscopy). Their clinical data are summarized in Table 1. The study took place in our research laboratory at the University of California, School of Optometry in Berkeley, California, from December 2004 to December 2009.

**Table 1:** Clinical profile of amblyopia.

Obs	Age (y)	Ethnicity	Gender	Eye	Refractive Error	Snellen VA Crowded (Isolated)	Cover Test (Distance)	SeA (arcsec)	Type of Amblyopia	Treatment Group		
										OT	VG-SM	VG-MOH
SS1	57.5	W	M	R	+0.50	20/240^+1^ (20/63^−2^)	R 5^Δ^ ExoT (6 m)	Failed (>400)	S			X
				L	+0.50/−0.75×180	20/16^−1^						
SS2	19.1	W	M	R	−1.50/−0.25×90	20/12.5^+1^	L 6^Δ^ ExoT (6 m)	Failed (>400)	S			X
				L	plano/−1.00×30	20/125^+2^ (20/63^−2^)						
SS3	18.9	C	F	R	−0.75	20/50^+2^ (20/32^−2^)	R 4^Δ^ EsoT (6 m)	Failed (>400)	S			X
				L	−0.25/−0.50×55	20/16^−2^						
SS4	22.2	P	M	R	+5.00/−2.25×5	20/16^+2^	L4^Δ^ EsoT (6 m)	400	S			X
				L	+5.50/−1.50×175	20/50^−2^ (20/32^+2^)						
SS5	18.0	C	F	R	plano/−0.50×95	20/16^+2^	L 4^Δ^ EsoT (6 m)	Failed (>400)	S			X
				L	−0.25/−0.50×50	20/50^−2^ (20/32^−1^)						
SS6	52.1	W	F	R	+1.25/−0.50×150	20/16^+1^	L>20^Δ^ ExoT (6 m)	Failed (>400)	S	X		X
				L	+1.00/−0.50×160	20/480^+1^ (20/95^+1^)						
SS7	28.8	W	F	R	−7.00	20/16^−1^	L 10^Δ^ EsoT (2 m)	Failed (>400)	S	X		X
				L	−7.00	20/190^−1^ (20/95^−1^)						
SS8	26.9	A	F	R	−0.50/−3.75×150	20/63^−2^ (20/25^−2^)	R 20–25^Δ^ EsoT (6 m)	Failed (>400)	S	X		X
				L	−2.00/−3.50×25	20/20^−2^						
SS9	45.5	W	F	R	+2.00	20/12.5^−1^	L 25^Δ^ ExoT (6 m)	140	S	X		
				L	+3.00/−0.75×95	20/40^+1^ (20/25^−2^)						
SS10	60.7	W	M	R	−1.50/−2.50×105	20/12.5^−1^	L 8^Δ^ ExoT	Failed (>400)	S	X		
				L	−3.00/−0.25×135	20/25^−1^ (20/25^−1^)	L 6^Δ^ hyperT (6 m)					
SA1	53.0	H	F	R	+2.00/−0.50×90	20/12.5^−2^	NMD (6 m)	200	A			X
				L	+4.25/−1.25×120	20/80^−2^ (20/63^−1^)						
SA2	15.3	W	F	R	−2.00/−175×155	20/32 (20/32)	NMD (6 m)	70	A			X
				L	+0.25/0.25×60	20/12.5						
SA3	24.3	W	M	R	−0.25	20/16^+2^	NMD (6 m)	30	A			X
				L	+1.75/−0.25×45	20/32^+2^ (20/32^+2^)						
SA4	19.2	W	M	R	+3.00/−1.00×25	20/25^−1^ (20/25^−1^)	4^Δ^ EsoP (6 m)	30	A			X
				L	+0.50/−0.75×150	20/12.5^−1^						
SA5	29.9	C	M	R	−1.50/−0.25×160	20/16^+1^	4^Δ^ ExoP (6 m)	200	A		X	X
				L	+0.75/−0.75×160	20/63^−2^ (20/50^+1^)						
SA6	26.7	W	F	R	+1.00	20/25^−2^ (20/25^+2^)	4^Δ^ EsoP (6 m)	Failed (>400)	A		X	X
				L	+0.25	20/12^−2^						
SB1	23.0	W	M	R	+3.50/−1.00×100	20/50^−2^ (20/32^−2^)	R 4^Δ^ EsoT (6 m)	Failed (>400)	S, A			X
				L	−0.50/−0.25×100	20/16^−1^						
SB2	52.4	W	M	R	plano/−1.00×125	20/16^+2^	L>40^Δ^ ExoT (1.5 m)	Failed (>400)	S, A		X	X
				L	−13.50/−2.00×140	20/400^+2^ (20/320^+1^)						
SB3	18.2	H	F	R	−0.50	20/16^−1^	L 8^Δ^ EsoT (6 m)	Failed (>400)	S, A	X		X
				L	+0.75/−0.75×70	20/63^−2^ (20/50^−2^)						
SC1	15.9	W	F	R	−4.25/−2.00×20	20/480^−2^ (20/380)	R 8^Δ^ EsoT (0.4 m)	Failed (>400)	S, C	X	X	
				L	−2.75/−0.75×150	20/20^+2^						

Abbreviations: (1) Ethnicity/Race. A, African; C, Chinese; H, Hispanic; P, Persian; W, White. (2) Cover test. ExoT, exotropia; EsoT, esotropia; HyperT, hypertropia; NMD, no movement detected. (3) SeA, stereoacuity. (4) Type of amblyopia. S, strabismic; A, anisometropic; C, deprivation (cataract). Note that participants' characteristics (such as age, gender, ethnicity, etc.) might not be balanced in each subgroup. (5) Treatment group. OT, occlusion therapy; VG, Video Game therapy; MOH, Medal of Honor Pacific Assault; SIM, SimCity Societies.

Participants were allocated into three intervention groups—two video-game treatment groups and one conventional occlusion therapy cross-over control group (Figure 1a). The first 10 enrolled patients participated in the action video game group, the subsequently enrolled three patients participated in the non-action videogame group, and then another seven patients were recruited in the cross-over intervention group of which participants were allowed to choose between the two types of video games (MOH: *n* = 4; SIM: *n* = 1, SC1) in phase 2. The two video games used were Medal of Honor Pacific Assault and SimCity Societies (Electronic Arts, Inc.). Since there has been no previous clinical evidence indicating that video games can modify vision in adult amblyopia in any way, in this pilot trial we decided to recruit participants for the video game treatment groups in the beginning, in order to evaluate the feasibility of this treatment approach. It is important to note that the participant allocation was not based on the clinical characteristics of participants.

In the main experiments, participants were required to play the assigned video games in our research laboratory for 40 or 80 h (2 h/d) using the amblyopic eye, with the fellow eye occluded with a black eye patch. They were given full optical correction for the viewing distance. A battery of vision function tests listed below was used to examine the effects of video-game experience on amblyopic vision (Figure 1b). All visual stimuli were displayed on a 21 in flat Sony F520 monitor screen at 1800×1440 resolution and 90 Hz refresh rate. Not all participants completed every visual function testing (visual acuity, *n* = 20; positional acuity, *n* = 16; visual counting, *n* = 14; stereoacuity, *n* = 5). Those participants in the control experiment (OT group) were given a log sheet to keep track of the patching hours and the visual tasks performed during patching.

### Visual Function Assessments

#### Visual acuity

Two Bailey-Lovie logMAR letter charts (#4, #5), National Vision Research Institute of Australia 1978, were used in measuring visual acuity (Figure 2d). The calculation of percent improvement is based on MAR.

#### Positional acuity

The stimuli and methods are essentially identical to those used in our previous studies of perceptual learning in amblyopia [4],[6],[9] and are described briefly as follows. A three-alternative, forced-choice (3AFC) procedure was used to determine the position-discrimination threshold. As illustrated in Figure 3a, the visual task was to pick the misaligned pair of Gabor patch groupings out of three choices (top, middle, or bottom) [23]. Each grouping consisted of 8 Gabor patches, which were constructed to have 1/3 aspect ratio. The mean center luminance of the stimuli was 54.5 cd/m^2^, and the contrast of each Gabor patch was 99%. Positional noise to the Gabor patches was introduced by varying their vertical positions according to a Gaussian distribution function. The average offset of each jittered Gabor patch grouping was constrained to be 0 by uniformly shifting the eight patches. An offset cue was produced by randomly shifting the right Gabor patch grouping up or down. The stimulus size and spatial frequency were scaled in rough proportion to their visual acuity in the amblyopic eye by varying viewing distance from 0.5 m to 4 m (at 4 m: carrier SF, 10 cpd; Gaussian envelope SD, [H] 1.25 arcmin & [V] 3.75 arcmin; segment separation, 17 arcmin; patch separation, 10.65 arcmin). Note that both the amblyopic eye and the fellow sound eye were tested at the same viewing distance.

A modified interleaved staircase method was adopted to control the offset magnitude between the two Gabor patch groupings and track the individual thresholds. Trial-by-trial feedback was provided. Positional threshold was defined as the offset at which 66% correct responses (*d*′ = 1.1) were obtained on a Weibull function (800 trials for all four noise levels).

A positional averaging model was used to quantify the effects of external noise (*σ_e_*) on the threshold (*σ_th_*):
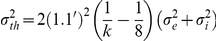
where *σ_i_* is the internal spatial distortion and *k* is the number of samples extracted. Sampling efficiency (*E*) was defined as:
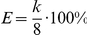
By measuring the thresholds at different external noise settings, *σ_i_* and *k* can be estimated with a least-square algorithm.

#### Spatial attention

Visual counting is used to examine spatial selective attention capacity of the brain to shift the focus of attention to individuate and attend to a number of objects at different locations in the visual field. A schematic diagram of the visual stimulus is illustrated in Figure 4a. Each trial started with a “bracket”-shaped fixation mark, indicating the upcoming stimulus location and area on the screen. A number (*N*) of black circular dots (0.5 cd/m^2^) was then presented for 200 ms against a gray background (42 cd/m^2^) , with Weber contrast of 99%. *N* ranged from 1–10 dots; the dots were randomly positioned in 10×10 square cells. The target stimulus was followed by a checkerboard pattern for another 100 ms, which was used to mask any after images of the dot stimuli. Each dot subtended 3 arcmin in diameter and was centered in its corresponding cell (6 arcmin×6 arcmin); the entire dot stimulus subtended 1° by 1° at a testing distance of 4 m. The distance between dots was at least two cells (edge-to-edge distance, ≥9 arcmin). The dot size was scaled with visual acuity (AE) by varying viewing distance (0.5 m, 1 m, 2 m, or 4 m), and therefore the dots displayed on the screen were very visible. The amblyopic eye and the fellow eye were both tested at the same viewing distance.

Observers were asked to enumerate the number of dots as quickly and accurately as they could. No feedback was given with respect to observers' responses. Counting threshold was taken as the midway between the upper and the lower free floating asymptotes (free floating) of a Weibull function as illustrated in Figure 4e. Each session block consisted of 100 trials, 10 trials for each *N*. The threshold reported for each observer was based on four blocks of measurement, i.e. a total of 400 trials. Response latency was measured using the time it took the observer to say the number into a microphone. Data acquisition of observers' voice responses was performed by an analog-to-digital converter (Measurement Computing Corporation, PCI-CTR05 board).

#### Stereoacuity

Randot Stereotest, Stereo Optical Co., Inc., was used to measure stereoacuity with wearing polarizing viewer (Figure 5a).
